# The *O*-Linked Glycome and Blood Group Antigens ABO on Mucin-Type Glycoproteins in Mucinous and Serous Epithelial Ovarian Tumors

**DOI:** 10.1371/journal.pone.0130197

**Published:** 2015-06-15

**Authors:** Varvara Vitiazeva, Jayesh J. Kattla, Sarah A. Flowers, Sara K. Lindén, Pushpa Premaratne, Birgitta Weijdegård, Karin Sundfeldt, Niclas G. Karlsson

**Affiliations:** 1 Department of Medical Biochemistry, University of Gothenburg, Gothenburg, Sweden; 2 Department of Obstetrics and Gynecology, Institute of Clinical Sciences, Sahlgrenska Cancer Center, University of Gothenburg, Gothenburg, Sweden; Inserm U995-Université de Lille, FRANCE

## Abstract

**Background:**

Mucins are heavily *O*-glycosylated proteins where the glycosylation has been shown to play an important role in cancer. Normal epithelial ovarian cells do not express secreted mucins, but their abnormal expression has previously been described in epithelial ovarian cancer and may relate to tumor formation and progression. The cyst fluids were shown to be a rich source for acidic glycoproteins. The study of these proteins can potentially lead to the identification of more effective biomarkers for ovarian cancer.

**Methods:**

In this study, we analyzed the expression of the MUC5AC and the *O*-glycosylation of acidic glycoproteins secreted into ovarian cyst fluids. The samples were obtained from patients with serous and mucinous ovarian tumors of different stages (benign, borderline, malignant) and grades. The *O*-linked oligosaccharides were released and analyzed by negative-ion graphitized carbon Liquid Chromatography (LC) coupled to Electrospray Ionization tandem Mass Spectrometry (ESI-MS^n^). The LC-ESI-MS^n^ of the oligosaccharides from ovarian cyst fluids displayed differences in expression of fucose containing structures such as blood group ABO antigens and Lewis-type epitopes.

**Results:**

The obtained data showed that serous and mucinous benign adenomas, mucinous low malignant potential carcinomas (LMPs, borderline) and mucinous low-grade carcinomas have a high level of blood groups and Lewis type epitopes. In contrast, this type of fucosylated structures were low abundant in the high-grade mucinous carcinomas or in serous carcinomas. In addition, the ovarian tumors that showed a high level of expression of blood group antigens also revealed a strong reactivity towards the MUC5AC antibody. To visualize the differences between serous and mucinous ovarian tumors based on the *O*-glycosylation, a hierarchical cluster analysis was performed using mass spectrometry average compositions (MSAC).

**Conclusion:**

Mucinous benign and LMPs along with mucinous low-grade carcinomas appear to be different from serous and high-grade mucinous carcinomas based on their *O*-glycan profiles.

## Introduction

Epithelial ovarian cancer (EOC) is the most lethal gynecological malignancy. This is mainly due to the fact that most of the EOC cases are diagnosed in late stages, when the 5-year survival rate is less than 37% [1]. Ovarian cancer is a relatively asymptomatic disease and as a result the detection of the EOC in earlier stages is a challenging task. Epithelial ovarian neoplasms are classified by histology into serous, mucinous, endometrioid, clear-cell and transitional or Brenner tumors [2].

Currently, Cancer Antigen (CA) 125 is the most used biomarker for ovarian cancer [3, 4]. A disadvantage of CA125 is its low sensitivity to ovarian carcinomas in stage I, where only 50% of patients have an increased level of CA125. Moreover, increased level of CA125 can also be detected in patients with endometriosis, pregnancy, uterine myomas, acute and chronic salpingitis and pelvic inflammatory disease [5, 6]. Of even greater concern is that CA125 and the more recently approved biomarker human epididymis 4 (HE4) [7] have limited diagnostic and prognostic value with the second most common type of EOC, mucinous ovarian cancer. Hence, mucinous ovarian tumors have a different biomolecular background that needs to be explored in order to improve diagnostics and treatment.

Nonetheless, CA125 is currently the best known biomarker for ovarian cancer, although over the last decade, several studies have been done in an attempt to find markers that can complement, improve or replace CA125 [7–10].

CA125 was identified as the transmembrane mucin MUC16 at the beginning of this century. As described in the literature, different types of epithelial cancer cells express different types of mucin glycoproteins on their surfaces [11–13]. Hence, it could be hypothesized that understanding differential mucin expression may lead to the identification of more effective biomarkers for the detection of various ovarian cancers and for following treatment progression.

Up to date, twenty one mucins are known. They can be subdivided into two groups: secreted mucins such as MUC2, MUC5AC, MUC5B, MUC6 and membrane bound mucins such as MUC1 and MUC16 [14] that could also be shed from the plasma membrane. Mucins are heavily glycosylated high-molecular mass proteins expressed by epithelial cells. The glycosylation can contribute up to, or even greater than 80% of the total molecular weight of glycoproteins. These mucin-type glycoproteins are predominantly decorated by *O*-linked and to lower extent also by *N*-linked oligosaccharides.

*O*-glycosylation is initiated by the attachment of GalNAc to serine or threonine residues of the apomucin protein by a family of GalNAc polypeptide transferases [15, 16]. Further extension by Galβ1- or GlcNAcβ1- at the *O*-3 position and/or by GlcNAcβ1- at the *O*-6 position followed by elongation by type 1/ type 2 (Galβ1→3/4GlcNAcβ1→) units are carried out by a series of Gal and GlcNAc transferases. Termination by sialyl- and fucose transferases generate epitopes such as sialyl Lewis a/x, blood group antigens A, B, O/H and Lewis a/b and Lewis x/y-type epitopes (Table 1) [15, 17]. Altered mucin glycosylation due to regulation of one or more of the glycosyltransferases together with ectopic expression of mucin-type glycoproteins is a hallmark of epithelial cancer [18–20]. A combination of both glycosylation and mucin expression may provide an alternative pathway to differentiate between ovarian cancer types.

**Table 1:** Examples of oligosaccharide epitopes expressed by mucin-type glycoproteins.

Epitope	Structure
Core 1	Galβ1→3GalNAcα1→
Core 2	Galβ1→3(GlcNAcβ1→6)GalNAcα1→
Core 3	GlcNAcβ1→3GalNAcα1→
Blood group O/H	Fucα1→2Galβ1→
Blood group A	GalNAcα1→3(Fucα1→2)Galβ1→
Blood group B	Galα1→3(Fucα1→2)Galβ1→
Lewis a	Galβ1→3(Fucα1→4)GlcNAcβ1→
Lewis x	Galβ1→4(Fucα1→3)GlcNAcβ1→
Lewis b	Fucα1→2Galβ1→3(Fucα1→4)GlcNAcβ1→
Lewis y	Fucα1→2Galβ1→4(Fucα1→3)GlcNAcβ1→

We have previously shown that ascites fluid from serous ovarian cancer contains highly sialylated and sulfated mucin-type molecules [21]. In this report, we move closer to the site of cancer by studying the *O*-linked oligosaccharides from acidic glycoproteins secreted into cyst fluids by mass spectrometry. The objective was to identify differences in mucin expression and glycosylation between mucinous and serous type ovarian cancer of various stages and grades in order to provide a pathway, which could lead to the discovery of an effective biomarker for mucinous ovarian cancer.

## Materials and Methods

### Clinical samples

Ovarian cyst fluids (n = 19) and tumor tissue (n = 16) from women with ovarian tumors of mucinous and serous histology were chosen from our ovarian tumor biobank (Table 2). Patients were diagnosed by transvaginal sonography or computed tomography and admitted for surgical removal of the cyst by gynecologic oncology surgeons at Sahlgrenska University Hospital, Gothenburg, Sweden. Blood samples were taken after anesthesia but before surgery, and ovarian cyst fluids were collected after removal of the cyst from the abdomen. All samples were immediately put at 4°C for 15–30 min, centrifuged, and aliquoted into eppendorf tubes. The fluids were transferred to −80°C, 30–60 min after collection.

**Table 2:** Clinical and biochemical characteristics of ovarian cyst fluid and tissue samples obtained from patients with mucinous and serous ovarian tumors.

Sample ^№/^Name	Blood group	Ag	Pato-histology				Plasma CA125 U/mL	Immunohistochemistry on tissue slides									Secretor status
			Type		Grade	Stage		MUC5AC			MUC16			Blood group			
								Stained cells score	Intensity score	Cellular localization	Stained cells score	Intensity score	Cellular localization	Stained cells score	Intensity score	Cellular localization	
^1^M-B^a^ ^,^ ^b^	B	44	Mucinous	cystadenoma	Benign	NR	17	3	3	cytoplasmic	1	3	membranous	3	3	cytoplasmic	positive
^2^M-B^a^	A	54	Mucinous	cystadenoma	Benign	NR	11.8	-	-	-	-	-	-	-	-	-	positive
^3^M-LMP-IA^a^	B	43	Mucinous	cystadenoma	LMP	IA	57.7	1	2	cytoplasmic	0	0	NR	2	1(2)	cytoplasmic	negative
^4^M-LMP-IA^a^	A	57	Mucinous	cystadenoma	LMP	IA	8.4	1	2	cytoplasmic	1	2	membranous/ cytoplasmic	3	3	cytoplasmic	-
^5^M-LMP-IA^a^	A	41	Mucinous	cystadenoma	LMP	IA	22.8	3	3	cytoplasmic	1	1	cytoplasmic / membranous	3	3	cytoplasmic	-
^6^M-LMP-IIIA	O	60	Mucinous	cystadenoma	LMP	IIIA	106.4	-	-	.	-	-	-	0	0	blood vessels stained positive	negative
^7^M-Low-IA^a^ ^,^ ^b^	A	36	Mucinous	+ endometroid adenocarcinoma	Low	IA	49.7	3	3	cytoplasmic	0	0	NR	3	2(3)	cytoplasmic	positive
^8^M-Low-IA^a^	A	55	Mucinous	cystadenocarcinoma	Low	IA	11.7	3	3	cytoplasmic	0	0	NR	3	2(3)	cytoplasmic	positive
^9^M-Low-IIIC^a^ ^,^ ^b^	A	53	Mucinous	adenocarcinom + mullerian mix	Low	IIIC	141.6	-	-	-	-	-	-	-	-	-	-
^10^M-Low-IV	O	69	Mucinous	adenocarcinoma	Low	IV	195.5	2	1	cytoplasmic	2	2	membranous	3	2	cytoplasmic	-
^11^M-High-IIB^a^	A	56	Mucinous	adenocarcinoma	High	IIB	33.8	0	0	NR	1	3	membranous/ cytoplasmic	3	2(3)	cytoplasmic	negative
^12^M-High-IA^a^	O	68	Mucinous	cystadenocarcinoma	High	IA	20.7	2	1,2,3	cytoplasmic	3	3	cytoplasmic / membranous	3	2(3)	cytoplasmic	positive
^13^M-High-IB^a^	O	72	Mucinous	cystadenocarcinoma	High	IB	37.9	0	0	NR	3	3	membranous	3	1(2)	cytoplasmic	positive
^14^S-B^a^ ^,^ ^b^	A	58	Serous	papillary cystadenoma	Benign	NR	18	0	0	NR	3	2	membranous/ cytoplasmic	3	3	cytoplasmic	-
^15^S-B^a^	O	59	Serous	papillary cystadenoma	Benign	NR	7.9	-	-	-	-	-	-	-	-	-	-
^16^S-LMP-IA	O	82	Serous	papillary cystadenoma	LMP	IA	61.6	0	0	NR	3	3	membranous/ cytoplasmic	3	2	cytoplasmic	-
^17^S-LMP-IIIC	A	44	Serous	papillary cystadenoma	LMP	IIIC	184.6	1	2	cytoplasmic	3	2	membranous	3	2	cytoplasmic	-
^18^S-LMP-IA^a^	O	40	Serous	papillary cystadenoma	LMP	IA	255.8	-	-	-	-	-	-	-	-	-	-
^19^S-Low-IA^a^ ^,^ ^b^	O	45	Serous	papillary cystadenocarcinoma	Low	IA	21.9	-	-	-	-	-	-	-	-	-	-
^20^S-Low-IIIC^a^	B	84	Serous	papillary cystadenocarcinoma	Low	IIIC	2314.6	-	-	-	3	3	membranous	3	2(1)	cytoplasmic	-
^21^S-High-IIIC	O	64	Serous	papillary cystadenocarcinoma	High	IIIC	2005.7	0	0	NR	3	3	membranous/ cytoplasmic	3	2	cytoplasmic	-
^22^S-High-IV^a^	A	73	Serous	papillary adenocarcinoma	High	IV	6547.7	0	0	NR	3	2	cytoplasmic / membranous	3	2	cytoplasmic	-
^23^S-High-IIIC^a^ ^,^ ^b^	A	85	Serous	papillary cystadenocarcinoma	High	IIIC	121.6	-	-	-	-	-	-	-	-	-	-
^24^S-High-IA^a^	A	54	Serous	papillary adenocarcinoma	High	IA	129.8	-	-	-	-	-	-	-	-	-	-

^a^ The O-linked oligosaccharides from cyst fluid were analyzed by LC-ESI-MS^n^.

^b^ The cyst fluid sample was analyzed by proteomic analysis.

NR- means not relevant

^“^–”means not determined

### Sample characteristics

All tumors were examined by an experienced pathologist for diagnosis, histology, and grade (Table 2). The tumors were staged (I-IV) according to the International Federation of Gynecology and Obstetrics (FIGO) 2014 standards. CA125 was measured in blood and cyst fluid samples from all patients with ovarian disease using ELISA-CA125 (Cisbio Bioassays, Codolet, France) according to the manufacturer’s instructions.

### Ethics Statement

The study was approved by the local ethics committee at the University of Gothenburg, and each patient provided their informed written consent. Patient samples were collected, decoded and stored by dedicated staff. Ethics approval was obtained for the collection and biobanking of cystic fluid, ascites, ovarian tissue and plasma (Dnr: S348-02 and S445-08).

### Acidic glycoproteins

Ovarian cyst fluid samples (~0,5 mL) were diluted (10 times) with starting buffer (250 mM sodium chloride in 20 mM Tris/10 mM ethylenediaminetetraacetic acid (pH 7.5) and injected on a diethylaminoethyl fast flow (1 mL) column (GE Healthcare, Uppsala, Sweden). After loading (10 min), the columns were washed with 10 mL of the starting buffer and the retarded fraction was collected (10 mL) by increasing the sodium chloride concentration to 1.0 M. Proteins were precipitated with ethanol (80% final concentration) at −20°C for 12 h. The obtained dried glycoproteins were resuspended in 3.5 M urea, dot blotted onto polyvinylidene fluoride (PVDF) membranes and stained with Direct Blue 71.

### Release of *O*-linked oligosaccharides

*O*-Linked oligosaccharides were released from PVDF membranes by reductive β-elimination as previously described [22] and analyzed by LC-ESI-MS^n^ using a 10 cm × 250 μm I.D. column, packed in house with 5 μm porous graphitized carbon particles (Thermo Scientific, Waltham, MA) and a linear gradient of 0 to 40% acetonitrile solution in water, containing 10 mM ammonium bicarbonate over 40 min with a static split flow rate of 250 μl/min bringing down the on-column flow rate to 5–10 μl/min.

Mass spectrometric data was collected in negative ion mode using a Thermo Scientific LTQ ion trap mass spectrometer (San Jose, CA) with an electrospray voltage of 3.5 kV, capillary voltage of −33.0 V, and capillary temperature of 300°C. The data were manually interpreted as described [23]. Structural assignment was performed by the comparison of MS^2^ spectra from isolated chromatographic peaks to structures identified in the UniCarb-DB glycomic database [24].

### LC-ESI-MS^n^ Identification of high-molecular-weight acidic glycoproteins

The acidic fraction from cyst fluids were reduced (50 mM dithiothreitol, RT, 2 hr) and alkylated (125 mM iodoacetamide, 30 min, RT in dark) before the reaction was stopped (125 mM dithiothreitol). Samples (25 μl) were separated using NuPAGE 3–8% Tris-acetate gels (Life Technologies) and stained with Imperial Coomassie protein stain (Thermo Scientific).

Coomassie stained gel bands and gel areas of interest were excised, destained (50 mM ammonium bicarbonate), dried and digested with 300 ng sequencing-grade Lys-C (50 mM ammonium bicarbonate, 4 h, 37°C) followed by 500 ng trypsin (Promega, Madison, WI, USA) (overnight, 37°C). Samples were dried and resuspended in 50 mM ammonium bicarbonate with 0.2% formic acid for LC-ESI-MS^n^ analysis. Data was searched against the UniProt human protein database using X! Tandem with the Orbitrap predefined method including reversed sequences [25].

All tryptic peptides were analyzed by nano-flow reverse-phase LC-electrospray ionization LC-ESI-MS^n^ using an LTQ-Orbitrap XL mass spectrometer (Thermo Scientific) as previously described [26]. Briefly, 3 μL of each digest was injected into a precolumn (4 cm × 100 μm inner diameter) and analytical column (20 cm × 50 μm inner diameter) packed with ReproSil-Pur C18-AQ 3 μm resin (Dalco Chromtech, Stockholm, Sweden). A split flow rate of 100 nL/min was used. A two-step gradient was used as follows: 0–40% solvent B (70% acetonitrile with 0.2% formic acid) in 50 min, 40–100% solvent B in a further 20 minutes. After washing with 100% B, the column was equilibrated with 100% solvent A (aqueous 0.2% formic acid). Full MS scans were obtained in the Orbitrap at *m/z* 400–2000, 2 microscans, maximum ion injection time 500 ms, and a target value of 500,000, using the lock mass feature for internal calibration (*m/z* 445.1200). Six data dependent MS^2^ scans were acquired in the ion trap using collision induced dissociation fragmentation and normalized collision energy of 30, activation time of 30 and activation energy of 0.250. The RAW file format were converted to mzXML using the MassMatrix conversion tool [27].

### Secretor status

Secretor status was evaluated by sequencing the coding exon of *FUT2*.

Genomic DNA was extracted from ovarian tissue cells in Clinical Molecular Pathology, Sahlgrenska University Hospital. The *FUT2* exon was amplified with PCR and sequenced in the Gene analysis, Clinical Chemistry, Sahlgrenska University Hospital using primer: FUT2-sense: 5’—CCC CAT CTT CAG AAT CAC C—3’ and FUT2-antisense:5’—TAC TGG TGA CCA CGA AGA TG—3’(Invitrogen Life Technologies, Thermo Fisher, Waltham, MA). Individual's secretor genotype was defined as non-secretor, when the in *FUT2* c.385 A>T (p.Ile129Phe), c.428G>A (p.Trp143stop) and c.571 C>T (p.Arg191stop).

### Immunohistochemical analysis

Ovarian tumor tissues were processed for immunohistochemical staining according to the manufacturer’s instructions (Vector Laboratories, Burlingame, CA, Thermo Scientific).

Paraformaldehyde-fixed paraffin-embedded 4-μm sections of ovarian tissue were deparaffinized, rehydrated and subjected to an antigen retrieval procedure using antigen unmasking solution (Vector Laboratories). The tissue sections were incubated with mouse monoclonal antibody: Anti-MUC5AC [clone 45M1; 1:400; Abcam], Anti-MUC16 [clone X75; 1:2000; Abcam] and biotinylated *Ulex Europaeus* Agglutinin I (UEA I) (1:400; Vector Laboratories). Negative controls underwent a similar staining procedure, with the exclusion of primary antibody or lectin. After incubation with secondary antibodies or streptavidin, immunodetection was performed using UltraVision Quanto Detection System HRP (Thermo Scientific).

Antigen expression levels were semiquantified on the basis of the proportion of cells of the same type cells staining positive. Positively stained cells were evaluated by the brown color, its intensity and spread in the tissues using a light microscope Nikon Eclipse 90i (Nikon, Tokyo, Japan) at ×200 magnification. The proportion of positive tumor cells were scored from 0–3 (None = 0, <1/3 = 1, 1/3-2/3 = 2, >2/3 = 3). The intensity of staining was scored from 0–3 (None = 0, Weak = 1, Moderate = 2, Strong = 3). The scoring of staining intensity was performed in a blinded manner. Scores represent the average score of 0.5 cm^2^ tumor. Porcine gastric tissues were used as positive control for MUC5AC and UEA I.

### Mass Spectrometry Compositional analysis

Mass spectrometry average compositions (MSAC) of oligosaccharides were calculated as described previously [28]. Briefly, all structures identified by LC-ESI-MS^n^ and presented in S1 and S2 Tables were reduced to monccharide compositions (the number of hexose (Hex), N-acetylhexosamne (HexNAc), fucose (Fuc), sialic acid (NeuAc) residues and sulfate groups (S) in the structure). The monosaccharide compositions were multiplied by percentage intensity for each structure and summed over the entire sample giving the MSAC values.

### Statistical analysis

Due to the limited number of samples, our data did not follow a typical Gaussian distribution. The separation test between mucinous and serous ovarian cyst fluid samples was evaluated by descriptive statistics such as receiver operation characteristic (ROC) curves. The area under the ROC (AUC) curves were constructed, compared and used for cluster analysis. Heat plots were constructed as described previously [28]. All statistical analyses were performed using the R package, version 3.0.2.

## Results

### The *O*-glycome from ovarian cyst fluid differs between serous and mucinous ovarian cancer by the expression of blood group antigens

Alterations in sialylation and sulfation of *O*-linked oligosaccharides have been described as a consequence of malignancy [18]. In addition, the histological differences between serous and mucinous epithelial ovarian cancer makes it likely that there are not only malignant differences in the *O*-glycosylation but also differences depending on the sub-type of cancer. These variations may not only be seen in the tissue but could also be reflected in the adjacent cyst fluids. To investigate these potential differences, ovarian cyst fluid and/or tumor tissues were collected from patients with benign and malignant serous (n = 11) and mucinous (n = 13) epithelial ovarian tumors of different stages and grades (Table 2). The *O*-glycomes of highly acidic glycoproteins from eight of the serous and eleven of the mucinous ovarian cyst fluids were studied by analyzing released *O*-linked oligosaccharides by LC-ESI-MS^n^ (Table 2, S1 and S2 Tables).

**Fig 1:**
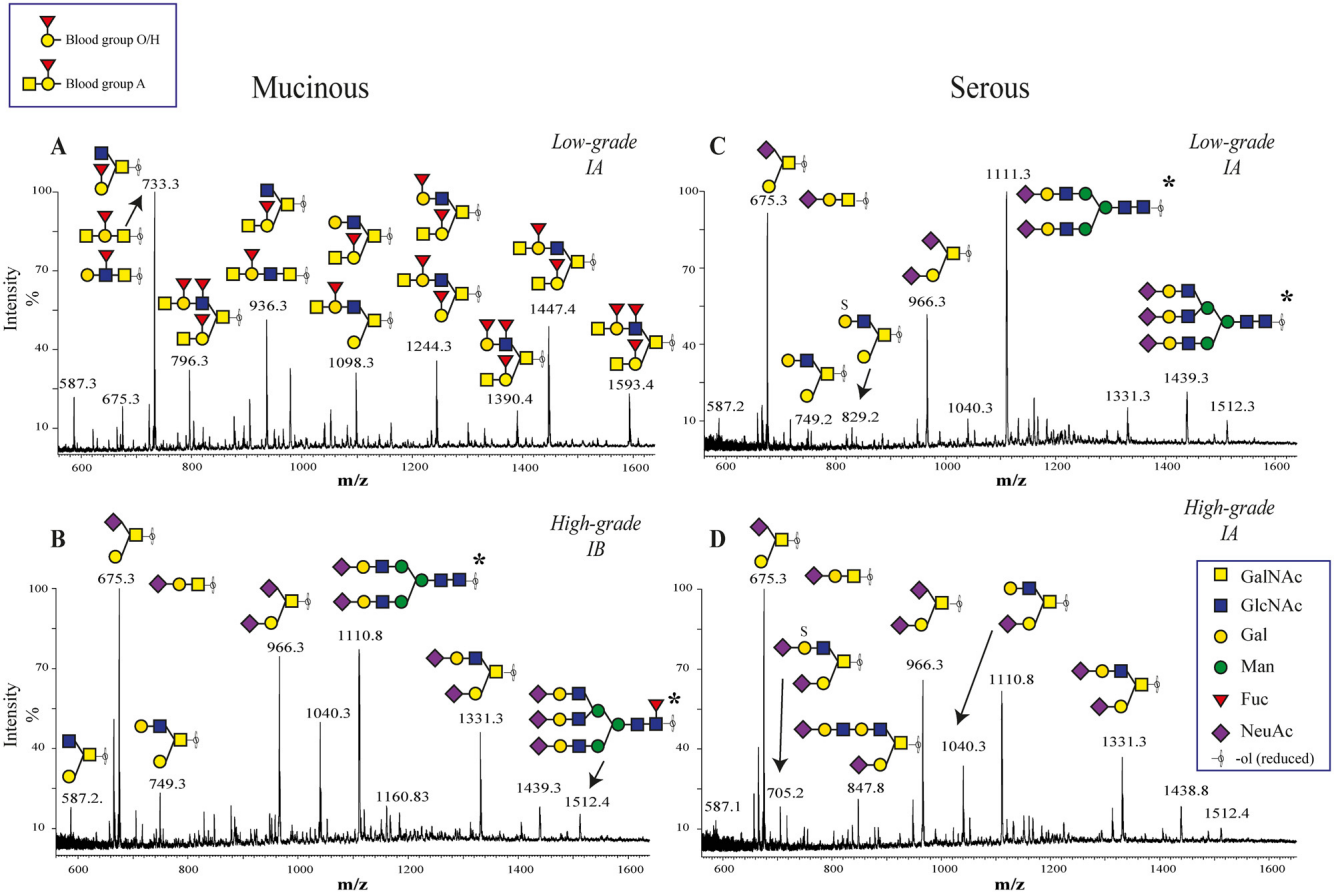
LC-ESI-MS spectra of *O*-linked oligosaccharides from mucinous and serous cyst fluids. Oligosaccharides was released by reductive β-elimination from acidic glycoproteins secreted into ovarian cyst fluids obtained from patients with high- and low-grade mucinous (A,B) and serous (C,D) OEC. Combined spectra of components eluted in the region 14–35 minutes in the chromatogram. Only major peaks identified are indicated. For all identified structures see S1 and S2 Tables. Fig 1A shows high abundant structures in low grade mucinous samples that are terminating with blood group O/H and blood group A, while structures in high grade mucinous (Fig 1B) as well as both low and high grade serous samples are terminating with sialic acid. *N-linked oligosaccharides also observed in the spectra.

The full scan spectra of *O*-linked oligosaccharides from the analyzed malignant serous (six samples of different grades and stages), but also four of the analyzed mucinous samples (one low malignant potential (LMP), three malignant high-grade carcinomas) cyst fluid samples were dominated by mono- and di-sialylated core 1 (Galβ1-3GalNAc-ol) and core 2 (Galβ1-3(Galβ1-4GlcNAcβ1–6)GalNAc-ol) (Fig 1B–1D). In contrast, the MS spectra of released oligosaccharides from the seven mucinous (two benign, two LMPs (borderline-type), three low-grade malignant) together with two serous benign samples were found to be much more heterogeneous (Fig 1A). Investigation of full scan and MS^2^ spectra showed that the *O*-linked oligosaccharides of these samples contained highly abundant structures containing fucose (Fuc). This indicated the presence of blood group ABO antigens and/or Lewis-type epitopes. MS^2^ fragmentation of the highly abundant fucose containing oligosaccharides from mucinous ovarian tumors demonstrated terminal sequences predominantly corresponding to blood group A (Fucα1-2(GalNAcα1–3)Galβ1-) blood group B (Fucα1-2(Galα-1-3)Galβ1-), and blood group O (Fucα1-2Galβ1-, also known as blood group H) epitopes. The mass spectrometric assignment that many of the structures were indeed blood group antigens (S1 Table) was strengthened by the match between the identified structures and the individual patients recorded blood group ABO status (Table 2).

### The secretor (Se) gene and the expression of blood group antigens in mucinous cyst fluid

**Fig 2:**
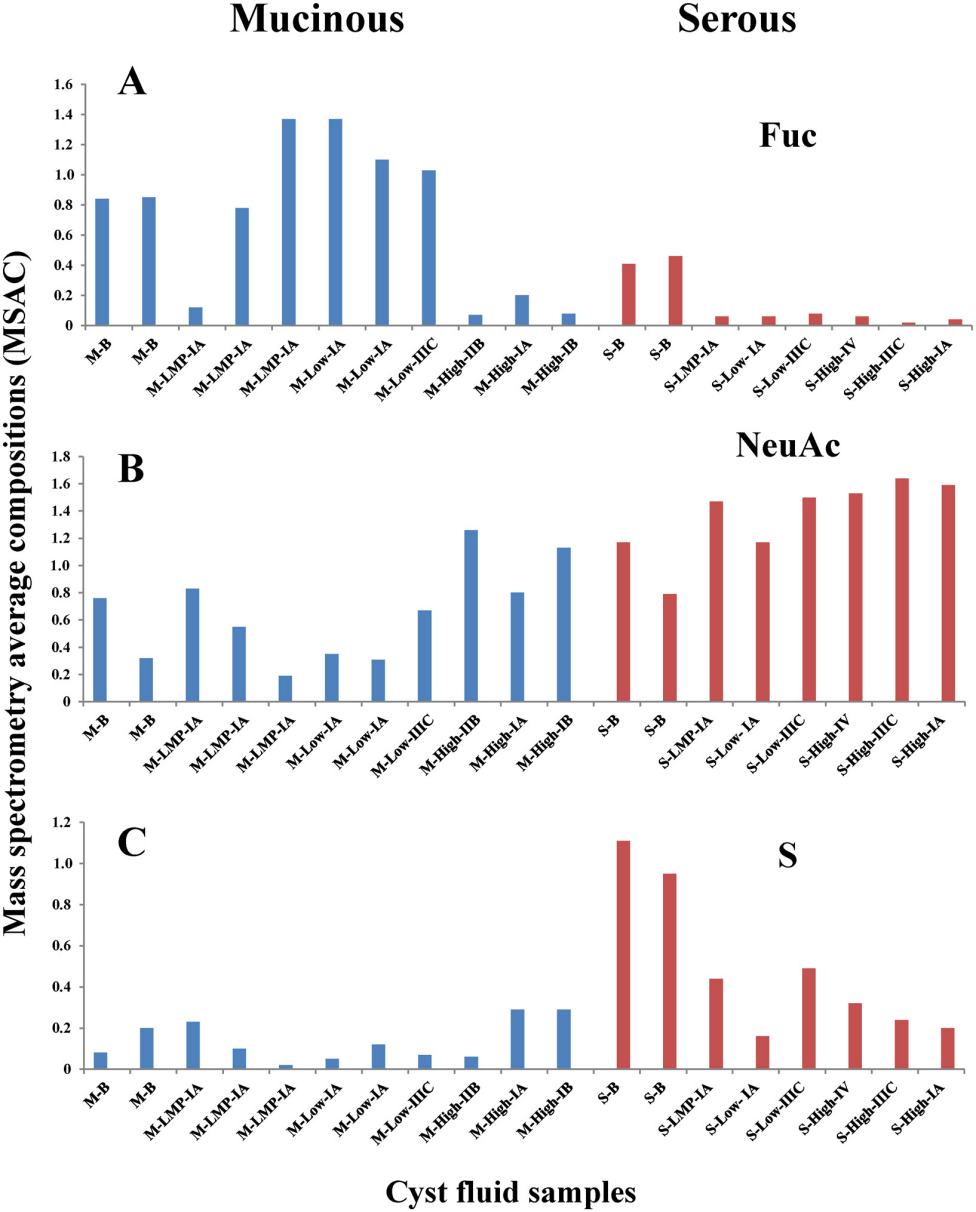
Difference in composition of *O*-linked oligosaccharides between mucinous and serous ovarian cancer. Diagrams demonstrating fucosylation (Fuc) (A), sialylation (NeuAc) (B) and sulfation (S) (C) levels of expressed *O*-linked oligosaccharides (MSAC) from mucinous and serous ovarian cyst fluids. Data (MSAC) is based on MS intensities of structures identified by LC-ESI-MS. Sample order corresponds to order in Table 2.

With the high expression of blood group antigens appearing to be the main differentiator between the mucinous and serous *O*-glycomes, our attention was drawn to mucinous patients’ cyst fluid that did not fit to this pattern (Fig 2A). It is known that about 20% of the population has a non-functional Fucα1–2 transferase *FUT2* (Se) gene, which is responsible for making the blood group O/H in secretion. *FUT2* is analogous to Fucα1–2 transferase *FUT1* and makes the same blood group O/H structure in the hemic system [29]. Hence, it was hypothesized that the low level of blood group antigens expression in mucinous ovarian cyst fluid could be due to the non-secretor status of the patients. For validation of this particular hypothesis, nine mucinous ovarian tumor samples were selected and subjected to pyrosequencing analysis of *FUT2* gene (Table 2). The analysis showed that 6 patients were homozygote or heterozygote carrying at least one copy of the most common functional *FUT2* allele with A at position 129, G at position 143 and C at position 191. The additional 3 were homozygotes for A at position 143, identifying them as non-secretors. LC-ESI-MS^n^ experiments on *O*-glycans indicated that, two of these patients have low expression of blood group antigens in their cyst fluids (^3^M-LMP-IA, ^11^M-High-IIB; Fig 2A). The third one (^6^M-LMP-IIIA; Table 2), where the corresponding cyst fluid was not available, was found by immunohistochemistry to have very low expression of fucosylated structures on the epithelial surface of the tumor tissue.

However, the non-functional Se gene could only partly explain the low amount of blood group antigens in cyst fluids in some mucinous sample. The data in Figs 1 and 2A also suggested that high-grade mucinous ovarian cancer cells express less blood group antigens compared to low-grade carcinoma, LMP and benign adenoma since the general fucosylation levels of *O*-glycans in samples were reduced. Furthermore, the analysis of *O*-glycosylation indicated that the high-grade mucinous glycosylation profile was similar to that from the malignant serous samples.

It should also be mentioned that the low expression of fucose found in four of the mucinous and most of the serous samples did not mean that these samples were devoid of blood group antigens. For instance, the full scan spectrum of serous papillary low grade cystadenocarcinoma stage IIIC sample (^20^S-Low-IIIC, S2 Table) showed low intense signal of [M-H]^-^ ions at *m/z* 1348 and 1057 indicating the expression of Fucα1-2(Galα1–3)Galβ1- epitope in agreement with blood group B of the patient (S2 Table).

### Fucosylation on epithelial ovarian cancer tissue

**Fig 3:**
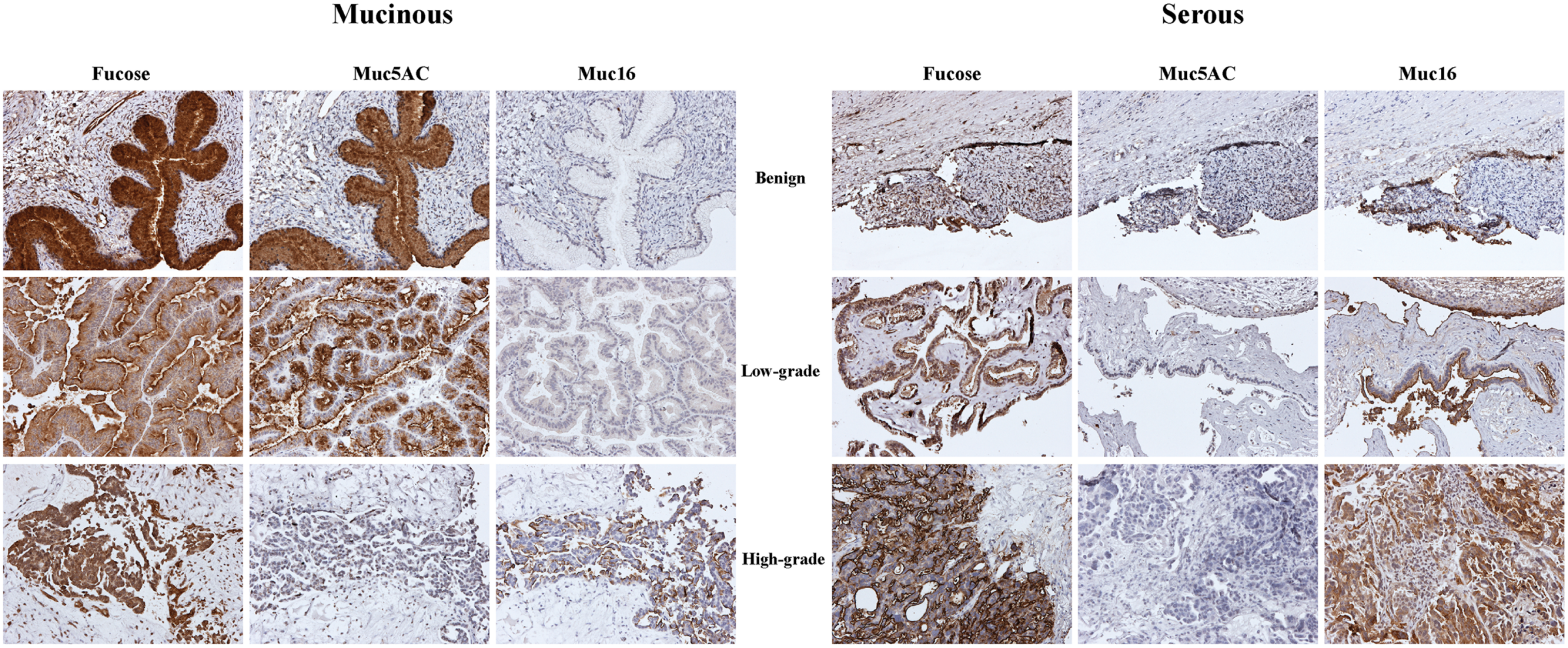
Tissue localization of α-fucose, MUC5AC (gel-forming mucin) and MUC16/CA125 (trans-membrane mucin) in benign, low-grade and high-grade serous and mucinous ovarian tumor cells. The figure shows the tissues from mucinous benign (^1^M-B), low-grade (^7^M-Low-IA), high-grade (^13^M-High-IB) as well as from serous benign (^14^S-B), low-grade (^20^S-Low-III), high-grade (^21^S-High-IIIC) tumors (Table 2). Tissues were stained with lectin (UEA I) against fucose, or mucin specific antibodies (anti-MUC5AC and anti-MUC16).

Since ovarian cyst fluids showed pronounced differences in *O*-glycosylation of mucin type glycoproteins, we were also interested in how the fucosylation (blood group ABO and/or Lewis type structures) found in mucinous cyst fluids were reflected in the corresponding cancer tissue. This was investigated by analyzing the reactivity of epithelial tissue to lectin, *Ulex europeus* Agglutinin I, recognizing fucosylated oligosaccharides (Fig 3; Table 2).

Cytoplasmic staining against fucose was noted in all examined ovarian tumors. However, in general, the serous tumors showed lower levels of expression of fucosylated structures compared to mucinous samples.

### MUC5AC is expressed in mucinous ovarian tumors

In order to determine the type of mucin proteins secreted into cyst fluids by the cancer tissue, proteomic analyses of high-molecular mass glycoproteins from acidic fractions were performed on a small sample set including mucinous and serous samples of benign cystadenoma, stage I and stage III adenocarcinomas. MUC5AC was detected in all mucinous samples and was not detected in any of the serous samples (Table 3). This observation was further substantiated by detection of MUC5AC in the tissue staining, where cytoplasmic staining was seen in the majority of mucinous ovarian tumors (Fig 3; Table 2). Lack of MUC5AC staining was observed for two high-grade mucinous adenocarcinomas, indicating a loss or lowered expression of MUC5AC in cancer cells with high differentiated grade. This observation is consistent with our LC-MS^n^ data detecting a relative reduction in *O*-linked oligosaccharides from high-grade mucinous cyst fluid samples. This finding suggests a reduction in the amount of MUC5AC secreted into cyst fluid in these samples rather than a lower antibody binding due to alteration in *O*-glycosylation.

**Table 3:** Mucins identified by mass spectrometry in cyst fluid samples obtained from patients with serous and mucinous ovarian benign tumors and stage I, III EOC.

Sample ^№/^Name	Mucin Identified	MW kDa	Protein Expectation value (log(e))	№ of peptides identified	Link
^1^M-B	Mucin-5AC (P98088)	526.3	-27.4	5/30	http://human.thegpm.org/thegpm-cgi/plist.pl?path=/gpm/archive/GPM32100041892.xml
^7^M-Low-IA	Mucin-5AC (P98088)	526.3	-255.5	32/186	http://human.thegpm.org/thegpm-cgi/plist.pl?path=/gpm/archive/GPM32100040512.xml
^9^M-Low-IIIC	Mucin-5AC (P98088)	526.3	-13.8	3/4	http://human.thegpm.org/thegpm-cgi/plist.pl?path=/gpm/archive/GPM32100041769.xml
^14^S-B	Mucin-5B (Q9HC84)	590.1	-58.5	8/11	http://human.thegpm.org/thegpm-cgi/plist.pl?path=/gpm/archive/GPM32100040515.xml
^19^S-Low-IA	No mucin detected				
^23^S-High-IIIC	Mucin-5B (Q9HC84)	590.1	-7	1/1	http://human.thegpm.org/thegpm-cgi/plist.pl?path=/gpm/archive/GPM32100040535.xml

**Mw-**Molecular weight of apoprotein obtained from protein database.

**Protein expectation value—log(e)**, the base 10 log of the expectation that the protein assignment was made at random. All identifications are of high confidence.

**№ of peptides identified**-the number of unique and total peptides used in the identification.

With the exception of one LMP tumor with low reactivity, all serous ovarian tumors showed an absence of immunoreactivity toward MUC5AC antibodies (Table 2). The tissue staining of MUC5AC and the fucose binding lectin staining were found to coincide in the mucinous ovarian tumor tissue indicating that the two molecules were secreted by the same cell and suggested that blood group antigens could be carried by MUC5AC in the tissue and in cyst fluid.

### MUC16 is poorly expressed by mucinous ovarian cancer cells

**Fig 4:**
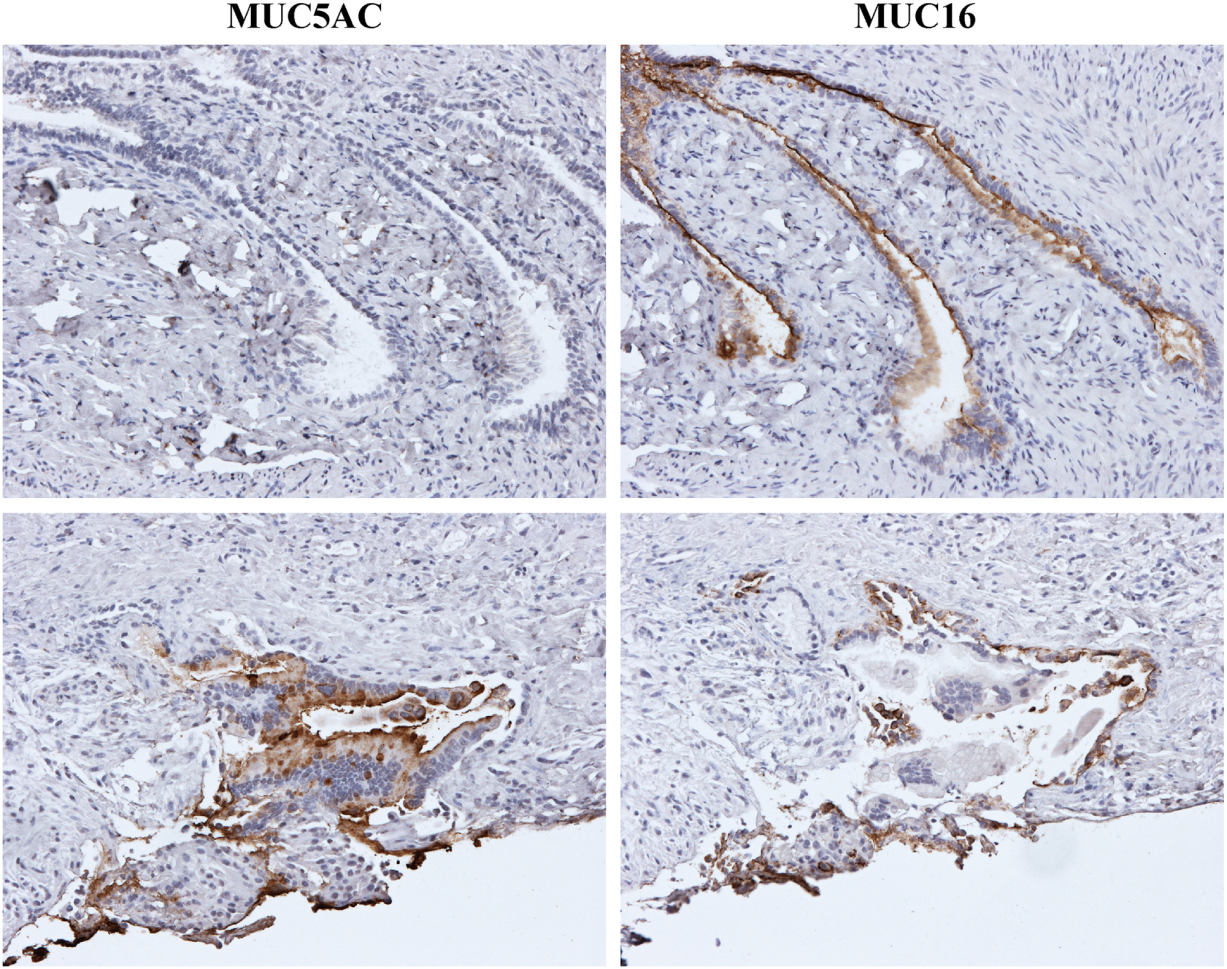
Localization of MUC5AC and MUC16 in mucinous adenocarcinoma. Serial sections from low-grade mucinous adenocarcinoma stage IV (^10^M-Low-IV) from (left-right) stained against MUC5AC and MUC16. The histological staining indicated that MUC5AC and MUC16 were expressed on discrete areas in the tumor.

Immunohistological staining with CA125/MUC16 antibody demonstrated the problem with utilizing this antigen for detection of mucinous ovarian cancer (Fig 3). All examined serous tumor tissues were MUC16 positive. In contrast, the level of staining of mucinous ovarian tumors varied, but tended to have a lower level of expression (Table 2). The CA125/MUC16 immunoreactivity was characterized predominantly by membranous staining but cytoplasmic staining was also present both in serous and mucinous samples. Two biopsies from two different mucinous ovarian carcinomas were positive for both MUC5AC and MUC16. However, the area expressing MUC16 did not express high levels of MUC5AC and vice versa (Fig 4). This observation suggested that mucinous ovarian tumors, expressing predominantly MUC5AC did not express, or expressed low levels of MUC16.

### Sialylation and sulfation of cyst fluid oligosaccharides

Since the secretor status highly influenced the fucose expression by epithelial ovarian tumor cells, two other oligosaccharide moieties for termination, sialic acid and sulfate, were investigated. The objective was to see how their appearance was correlated with the blood group antigens.

The Fucα1–2 transferase (*FUT2*) is responsible for synthesis of the Fucα1-2Galβ1-epitope, which is the common structure for all ABO blood group antigens. However, the galactosylated precursor for *FUT2* can also be elongated by α-(2,3)-sialyltransferase giving NeuAcα2-3Galβ1- terminal structure [30]. Hence, there could be a correlation between blood group expression and the level of sialylation of the *O*-glycome [31]. Sulfation, on the other hand, can terminate oligosaccharide elongation by the addition of a sulfate group to terminal non-reducing Gal, similar to the *FUT2* fucosyltransferase and sialyl transferases.

Alternatively it can act on internal residues, preferentially on the *O*-6 position of GlcNAc residues [32].

**Fig 5:**
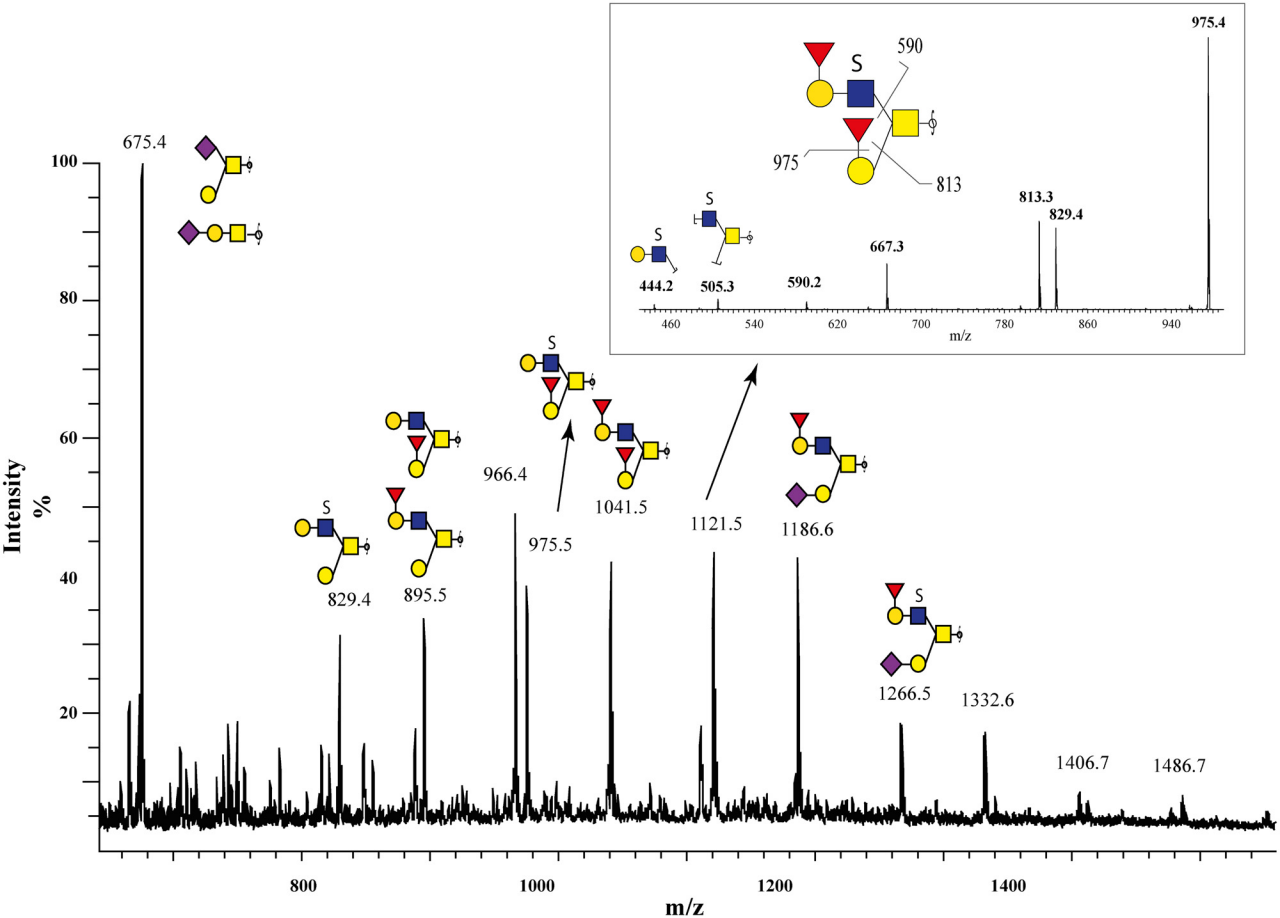
LC-ESI-MS spectrum of *O*-glycans with high sulfate content. Ovarian cyst fluid sample were obtained from patient with benign serous cystadenoma (^15^S-B) (A) and the LC-ESI-MS shows that it contains blood group O/H, sialylated and sulfated structures. Inserted panel shows MS^2^ spectrum of the [M—H] ion at *m/z* 1121.5 corresponding to the sulfated blood group H containing structure Fucα1-2Galβ1-(HSO_3_-)GlcNAcβ1-6(Fucα1-2Galβ1–3)GalNAc-ol (B). Proposed key fragments are indicated in the structure. Keys to the monosaccharide symbols see Fig 1.

The full scan spectra of the *O*-glycomes, illustrated that the expression of blood group antigens was not necessary in competition with the expression of sulfated glycans; the MS spectrum from one serous benign sample (^15^S-B) showed high abundant [M-H]^1-^ ions at *m/z* 829, 975, 1121 and 1266 corresponding to sulfated oligosaccharides (Fig 5A). Furthermore, the MS^2^ spectrum of [M-H]^-^ ion at *m/z* 1121 showed that it also carried two blood group O/H epitopes. Fragment ions at *m/z* 975, 813 and 590 corresponding to consecutive losses of the monosaccharide units in Fucα1-2Galβ1-3GalNAc-ol indicated that this was a blood group O/H core 2 type structure. The fragment ions at *m/z* 444 and 505 indicated that the sulfate group was located on the GlcNAc linked to the GalNAc-ol (Fig 5B), and both branches were shown to be terminated by a blood group O/H type epitope at their non-reducing end. Hence, it can be suggested that the sulfation of the GlcNAc unit is biosynthetically independent of both blood group ABO and sialylation.

The average amount of sulfate groups in serous benign samples was approximately 15–16% (S1 Table). However, the amount of sulfation in the *O*-glycome from mucinous benign samples was only up to approximately 6%. Therefore, it can be speculated that sulfation would also provide complementary information about mucinous/serous differentiation (Fig 2C).

### Serous epithelial ovarian cancer can be distinguished from mucinous low-grade ovarian cancer and benign mucinous and serous tumors

**Fig 6:**
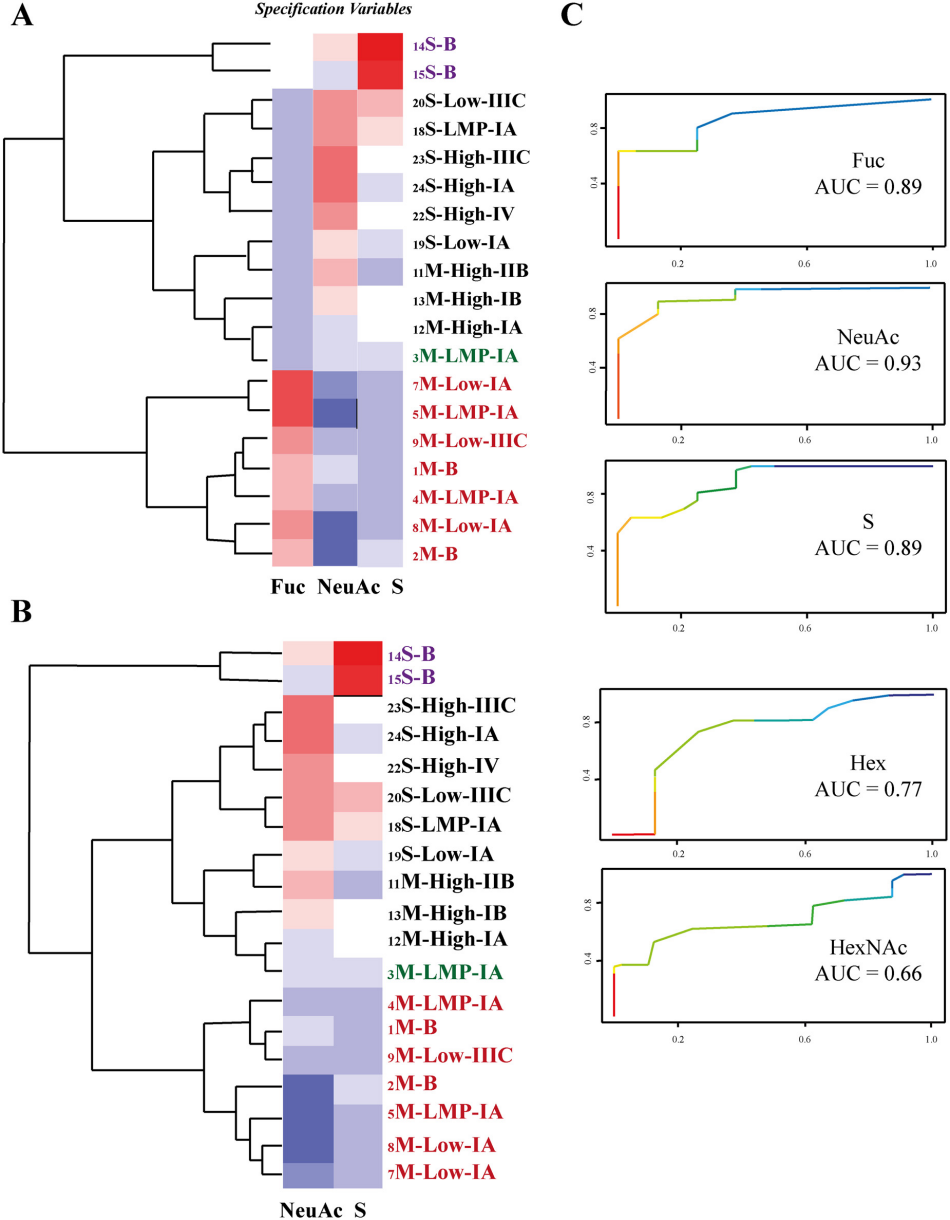
Hierarchical cluster analyses of *O*-glycans from serous and mucinous ovarian tumors. The clustering based on three variables Fuc, NeuAc and S (A) and also only the two variables NeuAc and S (B). The variables were evaluated using ROC AUC (C). The dendrograms (A, B) identify mucinous benign, LMPs and low-grade ovarian tumors as a distinct group (the group is marked by red color) separate from serous and high grade mucinous samples (the group marked in black). The exception is the LMP mucinous cyst fluid sample obtained from a non-secretor patient (green color). A separate cluster is generated by the two serous benign samples (violet color).

As Fig 2A highlights, the mass spectrometry average composition (MSAC) of the fucose residue (Fuc) appeared to be a good parameter for distinguishing the *O*-glycomes from serous and mucinous tumors. In order to validate this finding, nonparametric ROC analyses were performed for all MSAC parameters: Hex, HexNAc, Fuc, NeuAc and S (Fig 6). This analysis showed that Fuc, NeuAc and S are the most prominent parameters to distinguish the serous and mucinous samples (Fig 6) whereas AUC of Hex and HexNAc had less predictive value. Base on this, the heat plot was constructed using only Fuc, NeuAc and S (Fig 6A). The resulting plot shows the separation of the samples into three groups by hierarchical clustering. The analysis of these clusters confirmed our observation that low-grade, LMP and benign mucinous samples can be separated from all serous EOC samples, whereas, the serous benign samples form a separate group. However, as expected, one exception was observed. One mucinous LMP sample from a non-secretor patient did not follow this differentiation pattern (Fig 6; sample is marked by green color).

A statistical analysis of linear correlation coefficients between Fuc, NeuAc and S variables showed a strong correlation between Fuc and NeuAc (R = 0.89), possibly due to the biosynthetic competition between fucosylation and sialylation of *O*-linked oligosaccharides.

The heat plot using only two variables Fuc and NeuAc gave an equally good separation of all samples into three groups as by using three parameters (Fuc, NeuAc and S) (Fig 6B).

## Discussion

In the present study we investigated the differences in *O*-glycosylation of acidic glycoproteins secreted into cyst fluids by serous and mucinous epithelial ovarian tumors by mass spectrometry. In addition, the variation of expression of secreted mucin-type glycoproteins was also analyzed by immunohistochemistry. Since epithelial ovarian tumors are commonly grown in cystic formations, cyst fluids are enriched with glycoproteins secreted by malignant epithelial cells [33]. Consequently, the analysis of cyst fluids could aid the study of cancer differentiation and progression leading to new ovarian cancer biomarkers in order to improve early detection and the survival rate.

Our immunohistochemistry results showed that mucinous ovarian tumor cells were associated with a high level of expression of gastric-type mucin MUC5AC (gel-forming mucin), whereas the serous ovarian tumor cells did not show any substantial staining with the MUC5AC antibody. These results were in agreement with a previous immunohistochemistry study showing that none of 37 analyzed serous ovarian tumors expressed MUC5AC on their surfaces [11]. Although in another study, Giuntoli et. al showed mRNA expression of MUC5AC in both mucinous and serous ovarian cancer tissues by Northern blotting analyses [12]. They also found that transformed, nonmalignant ovarian epithelial cell lines expressed both *MUC5AC* and *MUC1* (membrane bound mucin) genes [12]. However, mucin expression and secretion are dynamic processes and direct correlation between mRNA level and the level of secreted protein is not always observed [34].

In the present study, MUC5AC was the only mucin-type glycoprotein that was identified by proteomic analyses of acidic high-molecular weight proteins obtained from three mucinous ovarian cyst fluids (benign cystadenoma, low-grade stage I and low-grade stage III adenocarcinomas). Although our data does not exclude the presence of other mucins in cyst fluids, it can be suggested that MUC5AC is the most abundant mucin-type glycoprotein in mucinous cyst fluids. The immunohistochemical staining showed some interesting features pointing to differentiation in expression of MUC5AC according to tumor cell differentiation grade. The expression of MUC5AC in the high-grade mucinous tumor cells seemed to be lower compared to benign, LMP and low-grade mucinous adenocarcinomas.

Consistent with previous observations [5, 10, 35], mucinous ovarian tumors in our study did not show a high level of expression of CA125/MUC16 neither on the tissue nor in the blood. Serous ovarian carcinomas with late stages III and IV displayed the highest level of CA125 in plasma (^21^S-High-IIIC—2005.7U/ml, ^22^S-High-IV—6547.7U/ml) whereas the level of CA125 in plasma from mucinous ovarian carcinomas at stages III and IV were much lower (^9^M-LowIIIC-141.6U/ml, ^10^M-Low-IV-195.57U/ml).

The present data indicated that serous and mucinous benign adenomas, mucinous LMP adenomas and mucinous low-grade carcinomas have a high level of fucosylation due to blood groups and Lewis-type epitopes, which were less abundant in the high-grade mucinous carcinomas and in all serous carcinomas. Our results suggested that the expression of fucosylated epitopes in mucinous tumors could be associated with differentiation grade. Welshinger et.al did not find a significant difference in survival among patients depending on reactivity of cancer tissue to antibody against blood groups antigens [36]. However, the majority of patients in that study had advanced diseases (stage III and IV) with differentiation grade 2 and grade 3 (high-grade differentiation). These data are consistent with our study that indicated the low expression of fucosylated glycoforms in all serous and high-grade mucinous carcinomas.

Expression of several gel-forming mucins has been described for mucinous ovarian tumors such as MUC2, MUC5AC and MUC5B [11, 12, 37–40]. In our study, the ovarian tumors, which showed a high level of expression of blood group antigens in cyst fluid glycoproteins, also revealed a strong reactivity towards the MUC5AC antibody. Thereby, the expression of fucosylated *O*-linked oligosaccharides could be related to MUC5AC expression. The *O*-linked glycomes of acidic glycoproteins from cyst fluids were found to be similar to oligosaccharide structures from a previously defined *O*-glycome of gastric mucins MUC5AC and MUC6, which were isolated from gastric tissues of healthy humans [41]. However, the *O*-glycome from mucins described in that study, did not reveal any detectible level of sulfated *O*-glycans, while the sulfation level in the mucinous *O*-glycome analyzed in our study was found to be between 0.3% and 6% (S1 and S2 Tables).

The LC-ESI-MS profiles of *O*-linked oligosaccharides derived from cyst fluids of serous ovarian and high-grade mucinous EOC indicated very low expression of fucosylated epitopes. The cyst fluid *O*-linked oligosaccharides resembled *O*-linked oligosaccharides obtained from ovarian cancer ascites with its high level of sulfation and sialylation [21], and indicated a direct link between ascites and cystic fluid *O*-linked glycosylation. Except for the sulfation level, the *O*-glycome from ovarian cancer ascites as well as the *O*-linked oligosaccharides from cyst fluids from all serous and high-grade mucinous EOC were found to be similar to *O*-linked oligosaccharides from glycoproteins present in human plasma, which predominantly contain the short mono- and di-sialylated core 1 and core 2 structures [42]. The proteomic study of ovarian cyst fluids also showed their high resemblance to serum [33]. This indicated that *O*-glycomes from serous and high-grade mucinous EOC may have a substantial contribution from surrounding plasma.

Interestingly, the highest level of sulfation was found in the serous benign cystadenoma. Generally, the sulfation level of *O*-glycome from serous carcinoma samples seem to be slightly higher than in highly fucosylated *O*-linked oligosaccharides.

The difference in *O*-glycosylation between mucinous low-grade and high-grade carcinomas could also be associated with alteration in glycosylation during cancer progression. In the last decade, quite a number of reports were published about aberrant glycosylation and its relation to epithelial cancer progression [30, 43]. For instance, the loss of expression of blood groups antigens has been observed in different types of cancer, such as a lung and breast cancer [44, 45]. However, epithelial cells such as colorectal epithelial cells that normally do not express ABO antigens on their surfaces show their expression in tumor formations [46, 47]. Pendu et al. proposed a working hypothesis that explains alterations in expression of blood groups antigens in epithelial cancers [30]. Briefly, they suggested that in the early stages of cancer, ABO antigens and Leewis b/y epitopes play an important role in epithelial cell resistance to apoptosis. This could explain their presence on epithelial cell surfaces at the initial stages of cancerogenosis even when they are not normally expressed. However, the loss of blood groups ABO at later stages increases cell motility and leads to metastasis [30].

Previously, glycan epitopes expressed by epithelial ovarian cancer cells were mostly studied by immunohistochemical analyses using monoclonal antibodies detecting Lewis-type structures (Le^a/x^, Le^y/b^, Sialyl-Le^a^ (sLe^a^)), Tn and STn and monoclonal antibodies against blood group antigens A,B and O/H [36, 48, 49]. In these studies, the high expression of the Le^y^ epitope was described for serous ovarian tumors, however, mucinous ovarian tumors were shown to react strongly to sLe^a^ antibody. Our LC-ESI-MS^n^ data of *O*-linked oligosaccharides derived from acidic glycoproteins secreted into ovarian cyst fluids showed that Le^y^ type epitopes were not present in the low molecular structures identified in this report. However monosaccharide compositions of larger detected structures did not exclude the possibility of Le^y^ epitopes among the low abundance high-molecular mass glycans (S2 Table). The presence of Le^y^ has also been shown to correlate with the expression of membrane-bound MUC1 and MUC16 mucins [49] as well as with expression of HE4 (Human Epididymis Protein 4) [50], which may explain the low abundance Le^y^ epitopes in *O*-linked oligosaccharides from secreted mucin-type glycoproteins.

Traditionally, epithelial ovarian tumors with different histologic sub-type and grade have been considered and treated as a single disease. However, the last published investigations have shown that they differ not only histologically, but also by gene and molecular expression, as well having a different clinical course and response to treatment [51–53].

Historically the treatment of cancer depends on the primary site, but it was suggested that treatments should be more dependent on the molecular characteristics of the tumor than on the organ of origin [54, 55]. Thus, to visualize the differences between glycosylation of ovarian cyst fluids, a hierarchical cluster analysis was performed as described by Hayes et al. [28]. Hierarchical clustering using three parameters (Fuc, NeuAc and S) or only two parameters (NeuAc and S) provided a differentiation of the samples that could be partly understood based on the assessment of grade and type but also provided additional suggestion for further stratification of the patients. One piece of clinical information that appeared to be important for the accuracy of predicting the mucinous ovarian cancer type based on the *O*-glycome from cystic fluid was the secretor status. This suggests that personalized diagnostics may be as important as personalized therapy.

In conclusion, the mucinous epithelial ovarian tumors can be distinguished from serous tumors by *O*-glycosylation profiles of mucin-type glycoproteins secreted into cyst fluids. Fucosylation as well as sialylation together with sulfation of *O*-glycans are important characteristics to differentiate the type of tumors. The presence of mucin MUC5AC in plasma has been reported for one epithelial cancer, cholangiocarcinoma [56].

Our next steps in the evaluation of mucins as a source for the new biomarkers for mucinous epithelial ovarian cancer will involve the development of serum assays specific to MUC5AC. The detailed study of MUC5AC *O*-glycosylation changes during mucinous ovarian cancer progression could lead to the development of assays recognizing both the apoprotein component and specific glycans of MUC5AC. For serous ovarian carcinomas a similar approach may also be possible to correlate specific *O*-glycan epitopes with the expression of MUC16/CA125 in order to improve the diagnostic value of this marker.

## Supporting Information

S1 Table LC-ESI-MS^n^ data on *O*-linked oligosaccharides obtained from mucinous ovarian tumor cyst fluids. (XLSX)

S2 Table LC-ESI-MS^n^ data on *O*-linked oligosaccharides obtained from mucinous ovarian tumor cyst fluids. (XLSX)
